# National Publication Productivity during the COVID-19 Pandemic—A Preliminary Exploratory Analysis of the 30 Countries Most Affected

**DOI:** 10.3390/biology9090271

**Published:** 2020-09-05

**Authors:** Simon M. Müller, Georg F. Mueller, Alexander A. Navarini, Oliver Brandt

**Affiliations:** 1 Department of Dermatology, University Hospital Basel, 4056 Basel, Switzerland; alexander.navarini@usb.ch (A.A.N.); o.brandt@usb.ch (O.B.); 2 Applied Computational Life Sciences, ZHAW Zurich University of Applied Sciences, 8400 Winterthur, Switzerland; muellgeo@students.zhaw.ch; 3 Pediatric Respiratory Medicine, Department of Pediatrics, Inselspital, University of Bern, 3010 Bern, Switzerland

**Keywords:** COVID-19, Sars-CoV2, publication productivity, research activity, pandemic

## Abstract

Background: The COVID 19 pandemic increased publication productivity enormously with numerous new COVID-19-related articles appearing daily, despite the fact that many health care workers in the partially overburdened national health care systems were faced with major challenges. Methods: In a cross-sectional, observational, retrospective study we compared and correlated 17 epidemiologic, health care system-related and health-economic factors from medical databases and intergovernmental organisations potentially influencing the COVID-19 and non-COVID-19 publication productivity between 1 January and 30 April 2020 amongst the 30 countries most severely affected by the pandemic. These factors were additionally correlated with the national pre-COVID-19 publication rate for the same pre-year period to identify potential changes in the general publication behaviour. Findings: COVID-19 and non-COVID-19 publication rates correlated strongest with access to and quality of health care (ρ = 0.80 and 0.87, *p* < 0.0001), COVID-19 cases per capita (ρ = 0.78 and 0.72, *p* < 0.0001), GDP per capita (ρ = 0.69 and 0.76, *p* < 0.0001), health spending per capita (ρ = 0.61 and 0.73, *p* < 0.0001) and the pre-COVID-19 Hirsch-Index (ρ = 0.61 and 0.62, *p* = 0.002 and <0.0001). Ratios of publication rates for “Cancer”, “Diabetes” and “Stroke” in 2020 versus the pre-year period were 0.88 ± 0.06, 1.02 ± 0.18 and 0.9 ± 0.20, resulting in a pooled ratio of 0.93 ± 0.06 for non-COVID-19 publications. Interpretation: There are marked geographic and national differences in publication productivity during the COVID-19 pandemic. Both COVID-19- and non-COVID-19 publication productivity correlates with epidemiologic, health care system-related and healtheconomic factors, and pre-COVID publication expertise. Countries with a stable scientific infrastructure appear to maintain non-COVID-19 publication productivity nearly at the pre-year level and at the same time use their resilience to produce COVID-19 publications at high rates.

## 1. Introduction

Despite massive measures taken worldwide, the consequences of the COVID-19 (i.e., SARS-CoV2 infection) pandemic are devastating. By the beginning of June 2020, the number of people infected had increased to more than 6.5 million and nearly 400,000 had died from this infection [1]. Not only clinicians focus on COVID-19 patients, but also the scientific medical community is making great efforts to identify effective treatment options and to disseminate new knowledge as urgently as possible for the benefit of all. This has resulted in an enormous increase in publication productivity with numerous new articles appearing daily in scientific journals, which is particularly surprising given the challenges faced by many health care workers in the partially overburdened national health care systems. We therefore sought to determine which factors could be decisive for the publication productivity during this crisis. Thus, we compared and correlated factors (Table 1) potentially influencing the COVID-19-related publication productivity between 1 January and 30 April 2020 amongst the 30 countries most severely affected by the pandemic. These factors were additionally correlated with the non-COVID-19 publication rate between 1 January and 30 April 2020 and the pre-COVID-19 publication rate for the same pre-year period to obtain information on potential changes in the general publication behaviour.

## 2. Methods

Selection of the potentially relevant factors was based both on previous non-COVID-19 articles addressing publication productivity of nations [10,11] and on an educated guess. Only data of highly reliable sources were retrieved to address/quantify these factors (Table 1). The sample size of 30 countries was based on King [11], intended to result in a representative selection of countries with high, intermediate and low (preliminary) publication output. To allow data collection despite the dynamic evolution of the pandemic, the ranking of the countries was defined on April 23 2020; however, COVID-19-related numbers (except for the case fatality rates) were updated on May 2 2020. The number of PubMed-listed publications was determined with the search term “COVID-19 AND country [affiliation]” and normalized by 1 million inhabitants. When using “SARS-CoV2” instead of “COVID-19”, search hits were markedly lower and yielded various duplicates with “COVID-19” search hits, thus we considered it an inferior search term for our analysis. Additionally, Scopus, Web of Science and Embase were searched for “COVID-19” and categorized by the countries affiliated. However, due to considerably lower search hits (Scopus *n* = 3048; Web of Science *n* = 998, Embase *n* = 3349), these databases were not considered for our analysis. For factors impossible to quantify precisely including “Research tradition/infrastructure”, “Research funding”, “Health care quality” approximative surrogate markers have been used (Table 1 [2,3,4,5,6,7,8,9]). Due to the dynamic changes of the national level of COVID-19 control measures, the stringency scores [4] were retrieved on 31 March and 30 April 2020, as we hypothesized that all of these factors could positively correlate with the number of PubMed-listed COVID-19-related publications. In the case of publications involving authors from institutions in different countries, each country affiliated was counted once. Due to the lack of reliable information, the number of medical researchers, medical universities and medical research funding per country could not be (directly) assessed.

To assess the non-COVID-19 publication rate between 1 January and 30 April 2020, we selected the topics “Cancer”, “Diabetes” and “Stroke”, as they exhibit a high year-round publication rate worldwide. The mean of the publications pooled from these three topics was normalized by 1 mio inhabitants (= “non-COVID-19 publication rate 2020”) and correlated with the same 17 factors mentioned above. Likewise, publication rates for “Cancer, “Diabetes” and “Stroke” between 1 January and 30 April 2020 were calculated (= “pre-COVID-19 publication rate”) and correlated with the factors listed in Table 1 (excluding the COVID-19-related ones). Spearman’s rank correlations were performed using SPSS 21 software (IBM Corporation: Amonk, NY, USA).

## 3. Results

**Figure 1:**
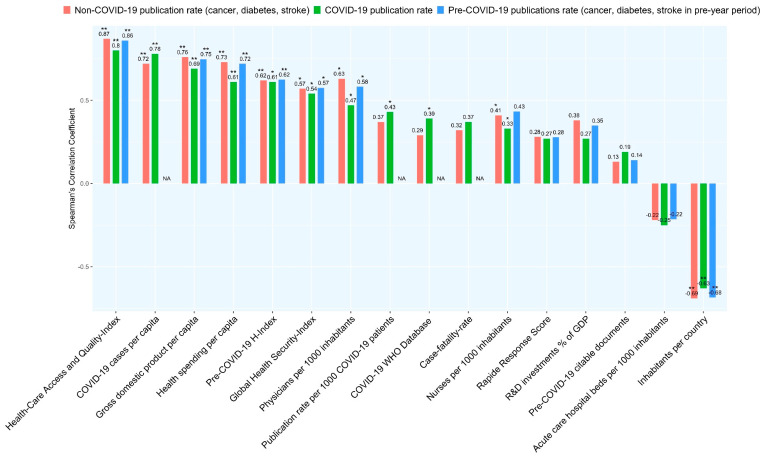
Correlations between COVID-19 publication rates and factors analysed. * *p*-value < 0.05, ** *p*-value < 0.0001.

Approximately two thirds (65.0%) of the analysed publications were affiliated with China, the USA, Italy, the UK or France, while Australia, African and Central American countries were not represented amongst the 30 nations with the highest COVID-19 rates. The results of the factors analysed varied considerably between countries (Table 2); the corresponding correlation coefficients are shown in Figure 1.

The Spearman’s correlation coefficients resulting from correlations between the COVID-19, nonCOVID-19 (i.e., publications on “Cancer”, “Diabetes” and “Stroke” published between 1 January and 30 April 2020 and pre-COVID-19 publication rates (i.e., publications on “Cancer”, “Diabetes” and “Stroke” published between 1 January and 30 April 2020) with the factors listed in Table 1 are shown in Figure 1 (exact *p*-values in Table A1). We found weak negative correlations between the non-COVID-19 publication rate and the national Stringency Index present on 31 March and 30 April 2020 (Rho (ρ) −0.344; *p* = 0.63 and ρ −0.314; *p* = 0.12). The COVID-19 publication rate neither correlated with these scores present on 31 March nor on 30 April 2020 (ρ −0.27; *p* = 0.13 and ρ −0.18; *p* = 0.37).

The COVID-19 and non-COVID-19 publication rate correlated positively (ρ 0.924; *p* < 0.001); the ratios of the publication rates for “Cancer”, “Diabetes” and “Stroke” 2020 vs. the pre-year period were 0.88 ± 0.06, 1.02 ± 0.18 and 0.9 ± 0.20 (Table A2), respectively, resulting in a pooled ratio of 0.93 ± 0.06 for non-COVID-19 publications.

## 4. Discussion

Our results indicate that the COVID-19 publication rate of the 30 countries most affected by the SARS-Cov2 pandemic by the end of April 2020 correlates positively with epidemiological factors (COVID-19 cases per capita, case fatality rate), health care system-relevant (number of physicians HAQ Index, Global Health Security Index) and economic factors (health care expenditure per capita, gross domestic product (GDP)), and, additionally, with the publication expertise (pre-COVID-19 H-index, COVID-19 trial database). In contrast, we have not detected a significant correlation between the COVID-19 publication productivity and the national level of stringency, number of acute care hospital beds and research and development (R&D) investment (as percentage of GDP). The correlation pattern we found for the COVID-19 publication rate was quite similar for the non-COVID-19-publication and, where applicable, also for the pre-COVID-19 publication rate, suggesting that it is not COVID-19 specific (Figure 1). This even held true when the two countries with the highest absolute (China, USA) and the highest normalized number (Singapore, Switzerland) of COVID-19 publications were excluded from the calculation.

It appears plausible that higher numbers of COVID-19 cases per capita and higher case fatality rates may drive publication productivity by (i) enlarging the pool of (severely ill) patients eligible for research, (ii) potentially increasing the number of contacts between COVID-19 patients and physicians and (iii) increasing the (social) pressure to help and thereby the motivation to publish COVID-19-related data. On the other hand, a higher burden on the health care system is likely to reduce resources for medical publications. The positive correlation between the number of physicians and publication productivity supports this assumption, as countries with very high (normalized) publication rates, such as Switzerland or Italy, have rather high numbers of physicians per 1000 inhabitants (no corresponding information was available for Singapore, which had the highest normalized publication rate). In this context, it would have been helpful to have information on the national proportion of (non-physician) researchers publishing on COVID-19, who are not directly involved in patient-care. Such a “division of labour” could explain why in some countries it was possible to “exploit” the pandemic for publications despite the high patient-related workload. Certainly, a well-established pre-COVID research and publication expertise may facilitate the latter. The high H-index (“pre-COVID-19 H-index”) from previous years we found for countries such as Italy, the Netherlands or UK supports the assumption that nations with a high COVID-19 publication rate have scientific infrastructures that enabled a high publication productivity despite high infection and case-fatality rates. The (weaker) positive correlation between registered COVID-19 trials and the current COVID-19 publication productivity may be rooted in the same pre-COVID publication and research expertise.

The extent of this utility of this pre-COVID expertise during the pandemic is presumably closely linked to health care-relevant and economic factors. The strongest correlation of the COVID-19 publication rate we found was with the HAQ-Index. This index is an approximation to assess health care access and quality based on 32 causes from which death should not occur in the presence of effective care. This “amenable mortality” [6] has been repeatedly used as an indicator for the overall performance of country’s health care system [6,12]. Except for Israel (HAQ-Index 85), all of the ten countries with the highest normalized publication rates had a very high HAQ-Index of ≥90 (with 100 being the maximum) indicating that higher quality of health care is associated with higher publication productivity. This obvious connection is further supported by the (weaker) correlation of the publication rate and the Global Health Security Index.

However, it remains speculative as to how the relationship between the quality of health care and the publication rate truly is, whether it is a coincidence or a causal association. This correlation may be confounded by economic factors, such as health spending per capita or GDP per capita, which also positively correlated with the publication rates. It is known from the Global Burden of Disease Study from 2016 that the HAQ-Index and the health spending per capita strongly correlate [6]. The differences in COVID-19 and non-COVID-19 publication rates between Italy and Spain are noticeable. Despite similarities in demographics, COVID-19 cases per capita, case-fatality rates, HAQ-Index and many other factors, the publication rate of Italy was more than 4 times higher for COVID-19 publications and approximately 1.5 times higher for non-COVID-19 publications. When considering the normalized factors, the major difference between these two countries may be the 32% higher health care spending per capita in Italy. It is, however, conceivable that the confinements established by Spain, which at that time were one of the strictest in the world, negatively affected scientific work and thus scientific output.

We also found a clear correlation between GDP and the publication rates for both COVID-19 and non-COVID-19 publications, but interestingly, only weak positive correlations between R&D investment (as percentage of GDP) and non-COVID-19 publication rate, but not with that of COVID-19 publications. The lack of correlation with the COVID-19 publication rate may be explained by the funding of non-medical investments irrelevant for health care-associated publications and possibly by the fact that the R&D figures used are from 2018 and thus from the period before the pandemic. This aspect may be of interest when analysing the publication rates in the next few years as Mendonca et al. [13] underlined the particular importance of the percentage of GDP spent on R&D for the publication rate by examining the impact of the financial crisis that started in 2008 on publication productivity. They were able to show that in countries severely affected by the financial crisis, publication output increased significantly slower in the subsequent years than in economically more stable nations. However, the influence of GDP on the number of scientific publications has been repeatedly studied with inconsistent results. While Cheng et al. [14] found clear correlations among publications in the field of rheumatology, and Liang et al. [15] when analysing arthroscopy publications, other authors were unable to demonstrate such a relationship [16,17].

Surprisingly, we found no correlation with the Rapid Response Score, a subcategory of the GHS-Index to assess the national emergency preparedness and response planning during an endemic [7]. It contains indicators of government responses overlapping with the ones present in the Stringency Index (e.g., structural/organizational preparedness, risk communication, travel restrictions), neither of which correlated with the publication rates in our analysis. This may possibly indicate that while the lockdown measures may be crucial to reduce COVID-19 transmissibility [18,19], they may not have a major influence on publication productivity by the timepoints of our assessments.

Likewise surprising was the fact that the publication rates of the three globally common diseases “Cancer”, “Diabetes” and “Stroke” between January 1 and April 30 2020 were unaffected for “Diabetes”, and decreased by only 10% compared to the same period in the previous year for the two other topics. Apparently, the level of productivity on these three non-COVID-19 topics could be nearly maintained despite the global predominance of the topic “COVID-19” and that many journals may have prioritized papers on COVID-19 to spread the scarce knowledge about this new virus rapidly. This further underlines the stability of the scientific infrastructure of scientifically very productive countries, in which, due to research units being shut down in many places, scientific work was only possible in home offices. However, these results must be interpreted cautiously because numerous of the non-COVID-19 publications had presumably been submitted before the pandemic. These results, together with our finding that most potential future studies have been registered with the WHO database by those nations that currently have the highest publication rates, make it likely that the majority of articles on COVID-19 will continue to originate from these countries.

Our study has some limitations: It is in the nature of exploratory studies using correlation analyses that they are not designed to detect confounding and cannot answer the type of relationship of the correlated factors. Thus, linear regression analyses might have produced somewhat different results. The factors we assessed and their corresponding rho-values may help to stratify this approach.

We noticed that many of the publications included in our analysis have international affiliations. Therefore, the focus on national publication rates we and others [10,11,14,17] have placed may be oversimplified as the globally connected research community cannot be adequately fitted into national terms. Furthermore, as the majority of Chinese medical publications are written in Chinese and are not listed in Pubmed, a presumably large number of publications on COVID-19 were not considered. Therefore, future analyses of COVID-19 publication productivity should ideally include publications in the Chinese language to obtain a more comprehensive view on this topic. Likewise, many future trials are not yet registered in the WHO database and it has been reported that up to 50% of registered studies are published with a latency of several years and some even remain unpublished [20,21]. Ultimately, our results cover just a four month period and 30 countries. In the two weeks following data analysis, the ranking of the countries most affected had already changed and countries not assessed by us are now amongst these top 30.

In conclusion, our analysis based on data from highly-trustful sources allowed us to rank 17 factors potentially influencing the national publication rates on COVID-19 and non-COVID-19 publications during the first 4 months of this pandemic. COVID-19 and non-COVID-19 publication rates correlated strongest with the access and quality of health care, the COVID-19 cases per capita, the GDP per capita, health spending per capita and the pre-COVID-19 H-Index. No correlations were found with the stringency of lock-down measures, the emergency preparedness for endemics, acute care hospital beds/1000 inhabitants, amongst others. Moreover, our results suggest that countries with a stable scientific infrastructure may be able to maintain non-COVID-19 publication productivity nearly at the pre-year level and at the same time use their resilience to produce COVID-19 publications at high rates.

## Figures and Tables

**Table 1:** National factors potentially influencing COVID-19-related publication productivity.

Factors	Assessed by	Source of Information
**Number of cases**	Absolute number Number/1000 inhabitants	JHU [1], UN [2]
**Severity of cases**	Case fatality rate	JHU [1]
**Non-COVID-19 publication productivity**	Number of PubMed-listed publications on “Cancer”, “Diabetes” and “Stroke” (sorted by countries of affiliations)	PubMed (www.pubmed.ncbi.nlm.nih.gov)
**Pre-COVID-19 research tradition/infrastructure**	Pre-COVID-19 H-index Pre-COVID-19 citable publications by country Number of PubMed-listed publications on “Cancer”, “Diabetes” and “Stroke” in the pre-year period	SCR [3] PubMed
**National COVID-19 control measures**	Stringency Index	Oxford COVID-19 Government Response Tracker [4]
**Research funding**	Pre-COVID-19 H-index, citable publications	SCR [3]
	Research and Development investments % of GDP	OECD [5]
	Health spending per capita	OECD [5]
**Number of physicians**	Physicians/1000 inhabitants	OECD [5]
**Number of acute beds**	Acute care hospital beds/1000 inhabitants	OECD [5]
**Health care quality**	Health care Access and Quality-Index (HAQ-Index)	GBDS [6]
	Global Health Security Index (GHS-Index)	NTI and JHU [7]
**Health care access**	Health care Access and Quality Index (HAQ Index)	GBDS [6]
**Health spending**	Annual spending per capita	OECD [5]
**Response to and mitigation of the spread of an epidemic**	Rapid Response» subcategory of Global Health Security Index (GHS-Index)	NTI and JHU [7]
**Gross domestic product (GDP)**	Gross domestic product (at purchasing power parity) per capita estimated for 2020 (USD)	IMF [8]
**Potential future clinical trials**	Number of currently registered COVID-19 trials	WHO COVID-19 Database (www.clinicaltrials.gov) [9]

**Table 2:** Overview of the 30 countries most affected by COVID-19 by the end of April 2020. For each country, the COVID-19 publications and non-COVID-19 publications between January 1 and April 30 2020 are listed, including the factors analyzed.

	COVID-19 Cases	Inhabitants Per Country	COVID-19 Cases Per Capita	COVID-19 Case Fatality Rate	PubMed-Listed COVID-19 Publications	PubMed-Listed COVID-19 Publications Per Mio Inhabitants	Non-COVID-19 Publications	Non-COVID-19 Publications Per Mio Inhabitants	COVID-19 Publication Rate Per 1000 COVID-19 Cases	Pre-COVID-19 H-Index	Pre-COVID-19 Citable Documents	Physicians Per 1000 Inhabitants	Nurses Per 1000 Inhabitants	Acute Care Hospital Beds Per 1000 Inhab	R&D Investments % of GDP	Health Spending Per Capita (USD)	Global Health Security Index	Health Care Access and Quality Score	Emergency Preparedness and Response Planning Score	GDP Per Capita	Registered COVID-19 Studies in WHO Database
**USA**	1,104,161	331,002,651	0.00334	0.05	1084	3.27	19,239	58.12	0.85	1498	3,683,931	2.61	11.74	2.40	2.83	10,586.80	83.5	89	79.70	67,426	217
**Spain**	213,435	46,754,778	0.00456	0.10	167	3.57	3379	72.27	0.49	609	411,210	3.88	5.74	2.40	1.24	2341.38	65.9	92	61.90	43,007	53
**Italy**	207,428	60,461,826	0.00343	0.13	918	15.18	6234	103.11	3.27	752	567,666	3.99	6.71	2.60	1.39	3427.81	56.2	95	47.50	41,582	78
**France**	167,305	65,273,511	0.00256	0.13	339	5.19	4651	71.25	1.45	810	594,400	3.37	10.80	3.10	2.20	4964.71	68.2	92	62.90	48,640	194
**Germany**	164,077	83,783,942	0.00196	0.03	256	3.06	6443	76.90	1.15	844	865,665	4.25	12.93	6.00	3.13	5986.43	66.0	92	54.80	55,306	38
**UK**	178,685	67,886,011	0.00263	0.13	643	9.47	6684	98.46	3.49	1004	990,889	2.85	7.80	2.10	1.71	4069.57	77.9	90	91.90	48,168	35
**Turkey**	122,392	84,339,067	0.00145	0.02	88	1.04	1302	15.44	0.55	284	230,057	1.87	2.07	2.80	0.96	1226.59	52.4	74	49.00	29,326	13
**Iran**	96,448	83,992,949	0.00115	0.06	187	2.23	1866	22.22	1.55	191	121,946	NA	NA	NA	NA	NA	37.7	72	33.70	17,831	26
**China**	83,959	1,439,323,776	0.00006	0.06	1991	1.38	22,337	15.52	19.79	440	104,4497	2.01	2.70	NA	2.19	688.00	48.2	78	48.60	20,984	98
**Russia**	124,054	145,934,462	0.00085	0.01	25	0.17	633	4.34	0.28	283	114,520	4.04	8.47	NA	0.99	1513.67	44.3	75	50.10	30,819	4
**Brazil**	92,202	212,559,417	0.00043	0.06	119	0.56	1980	9.32	1.97	404	278,872	NA	NA	NA	NA	1281.62	59.7	64	67.10	17,016	18
**Belgium**	49,517	11,589,623	0.00427	0.15	95	8.20	1533	132.27	1.34	603	175,380	3.08	10.96	5.00	2.76	4943.54	61.0	93	47.30	50,904	19
**Canada**	56,343	37,742,154	0.00149	0.04	298	7.90	5050	133.80	5.34	849	515,151	2.76	9.96	1.90	1.54	4974.33	75.3	94	60.70	52,144	30
**Netherlands**	39,989	17,134,872	0.00233	0.12	130	7.59	3392	197.96	2.31	752	355,369	NA	NA	2.90	2.16	5288.44	75.6	96	79.10	60,299	14
**Switzerland**	29,817	8,654,622	0.00345	0.05	186	21.49	2173	251.08	4.74	669	233,184	4.30	17.23	3.60	3.37	7316.60	67.0	96	79.30	67,557	18
**Portugal**	25,351	10,196,709	0.00249	0.04	44	4.32	895	87.77	1.27	313	67,008	NA	6.70	3.30	1.35	2861.38	60.3	86	67.70	34,935	5
**India**	37,336	1,380,004,385	0.00003	0.03	297	0.22	2950	2.14	9.47	372	336,884	0.78	1.50	NA	NA	208.77	46.5	41	52.40	9026	8
**Peru**	40,459	32,971,854	0.00123	0.03	15	0.45	105	3.18	0.62	176	9013	NA	NA	NA	NA	NA	49.2	64	51.70	15,398	0
**Ireland**	20,833	4,937,786	0.00422	0.05	61	12.35	765	154.93	2.68	361	59,293	3.18	12.16	2.80	1.15	4915.49	59.0	95	45.10	86,988	4
**Sweden**	21,520	10,099,265	0.00213	0.12	72	7.13	2313	229.03	3.44	624	22,5333	4.12	10.90	2.00	3.31	5447.11	72.1	95	62.80	55,988	7
**Austria**	15,558	9,006,398	0.00173	0.03	52	5.77	1246	138.35	2.08	454	120,055	5.18	6.85	5.50	3.22	5395.11	58.5	94	42.30	55,171	12
**Israel**	16,152	8,655,535	0.00187	0.01	83	9.59	1568	181.16	4.19	447	114,181	3.14	5.08	2.20	4.94	2779.66	47.3	85	39.90	40,336	9
**Saudi Arabia**	24,097	34,813,871	0.00069	0.01	76	2.18	843	24.21	4.99	191	47,503	NA	NA	NA	NA	NA	49.3	77	32.60	5,6912	2
**Japan**	14,305	126,476,461	0.00011	0.02	137	1.08	6636	52.47	9.12	619	857,878	2.43	11.34	7.80	3.26	4766.07	59.8	94	53.60	46,827	4
**Chile**	17,008	19,116,201	0.00089	0.01	20	1.05	351	18.36	1.11	228	35,875	NA	NA	2.00	0.36	2181.73	58.3	78	60.20	2715	2
**South Korea**	10,780	51,269,185	0.00021	0.02	117	2.28	810	15.80	9.26	371	269,058	2.34	6.91	7.10	4.53	3191.55	70.2	90	71.50	46,451	5
**Ecuador**	26,336	17,643,054	0.00149	0.05	6	0.34	49	2.78	0.38	149	17,681	NA	NA	NA	NA	NA	50.1	62	39.50	11,866	0
**Singapore**	17,548	5,850,342	0.00300	0.00	190	32.48	894	152.81	15.09	535	269,110	NA	NA	NA	NA	NA	58.7	91	64.60	105,689	7
**Poland**	13,375	37,646,611	0.00036	0.04	48	1.28	1446	38.41	3.69	519	627,632	NA	NA	4.80	NA	2056.36	55.4	82	47.50	35,651	9
**Pakistan**	18,114	220,892,340	0.00008	0.02	52	0.24	417	1.89	3.28	247	143,723	NA	NA	NA	NA	NA	35.5	38	38.70	6016	6

## Abbreviations

GBDS	Global Burden of Disease Study
GHS-Index	Global Health Security Index
HAQ-Index	Health care Access and Quality
JHU	Johns Hopkins University
IMF	International Monetary Fund
NTI	Nuclear Threat Initiative
OECD	Organisation for Economic Co-operation and Development
SCR	SCImago Country Ranking by SCOPUS
UN	United Nations
WHO	Word Health Organization

## Appendix A

**Table A1:** Correlations between COVID-19-, non-COVID-19- and pre-COVID-19-publication rate and factors analysed (rho (ρ)- and *p*-values).

Factors	Correlation with COVID-19 Publication Rate, rho	*p*-Values	Correlation with Non-COVID-19 Publication Rate (Cancer, Diabetes, Stroke), rho (ρ)	*p*-Values	Correlation with Pre-COVID-19 Publication Rate, rho (ρ)	*p*-Values
**Health Care Access and Quality Index**	0.80	<0.0001	0.87	<0.0001	0.86	<0.0001
**COVID-19 cases per capita**	0.78	<0.0001	0.72	<0.0001	NA	NA
**Gross domestic product per capita**	0.69	<0.0001	0.76	<0.0001	0.748	<0.0001
**Health spending per capita**	0.61	<0.0001	0.73	<0.0001	0.72	<0.0001
**Pre-COVID-19 H-index**	0.61	0.002	0.62	<0.0001	0.625	<0.0001
**Global Health Security Index**	0.54	0.002	0.57	0.001	0.575	0.001
**Physicians per 1000 inhabitants**	0.47	0.045	0.63	0.004	0.582	0.009
**Publication rate per 1000 COVID-19 patients**	0.43	0.032	0.37	0.124	NA	NA
**COVID-19 WHO Database**	0.39	0.042	0.29	0.082	NA	NA
**Case-fatality-rate**	0.37	0.154	0.32	0.071	NA	NA
**Nurses per 1000 inhabitants**	0.33	0.017	0.41	0.047	0.433	0.056
**Rapid Response score**	0.27	1.45	0.28	1.33	0.278	0.137
**R&D investments % of GDP**	0.27	0.241	0.38	0.089	0.348	0.122
**Pre-COVID-19 citable documents**	0.19	0.325	0.13	0.48	0.141	0.456
**Acute care hospital beds per 1000 inhabitants**	−0.25	0.28	−0.22	0.36	−0.215	0.364
**Inhabitants per country**	−0.63	<0.0001	−0.69	<0.001	−0.685	<0.0001

**Table A2:** The absolute numbers and percentage of the 2020/2019 ratio of the COVID-19- and the non-COVID-19-publication rates for Cancer, Diabetes and Stroke between January 1 and April 30 2020 and the pre-year period.

	Cancer 1.1.–30.4.19	Cancer 1.1.–30.4.20	Cancer Percentage 2020/2019	Diabetes 1.1.–30.4.19	Diabetes 1.1.–30.4.20	Diabetes Percentage 2020/2019	Stroke 1.1.–30.4.19	Stroke 1.1.–30.4.20	Stroke Percentage 2020/2019
**USA**	15,839	14,354	0.91	3503	3306	0.94	1776	1579	0.89
**Spain**	2529	2323	0.92	726	763	1.05	308	293	0.95
**Italy**	5218	4528	0.87	975	1153	1.18	609	553	0.91
**France**	4040	3551	0.88	675	688	1.02	482	412	0.85
**Germany**	5139	4570	0.89	1142	1160	1.02	767	713	0.93
**United Kingdom**	5023	4438	0.88	1516	1586	1.05	717	660	0.92
**Turkey**	1286	914	0.71	376	311	0.83	120	77	0.64
**Iran**	1533	1337	0.87	588	446	0.76	100	83	0.83
**China**	19,034	17,612	0.93	2999	3226	1.08	1483	1499	1.01
**Russia**	571	486	0.85	105	85	0.81	124	62	0.50
**Brazil**	1482	1369	0.92	443	465	1.05	183	146	0.80
**Belgium**	1267	1117	0.88	255	289	1.13	153	127	0.83
**Canada**	3686	3458	0.94	928	922	0.99	698	670	0.96
**Netherlands**	2698	2502	0.93	638	562	0.88	390	328	0.84
**Switzerland**	1603	1458	0.91	377	445	1.18	271	270	1.00
**Portugal**	659	618	0.94	164	194	1.18	106	83	0.78
**India**	2705	2008	0.74	922	778	0.84	206	164	0.80
**Peru**	76	59	0.78	21	34	1.62	7	12	1.71
**Ireland**	480	482	1.00	151	212	1.40	89	71	0.80
**Sweden**	1647	1459	0.89	619	590	0.95	254	264	1.04
**Austria**	908	888	0.98	219	224	1.02	139	134	0.96
**Israel**	1189	1047	0.88	364	355	0.98	164	166	1.01
**Saudi Arabia**	648	564	0.87	272	242	0.89	50	37	0.74
**Japan**	6128	5042	0.82	1166	1044	0.90	659	550	0.83
**Chile**	227	218	0.96	93	102	1.10	33	31	0.94
**South Korea**	702	583	0.83	141	145	1.03	102	82	0.80
**Ecuador**	35	27	0.77	13	12	0.92	8	10	1.25
**Singapore**	662	571	0.86	219	241	1.10	85	82	0.96
**Poland**	1276	1022	0.80	366	291	0.80	173	133	0.77
**Pakistan**	320	275	0.86	150	119	0.79	30	23	0.77
Mean			0.88			1.02			0.9
SD			0.06			0.18			0.20
